# Compound 15c, a Novel Dual Inhibitor of EGFR^L858R/T790M^ and FGFR1, Efficiently Overcomes Epidermal Growth Factor Receptor-Tyrosine Kinase Inhibitor Resistance of Non-Small-Cell Lung Cancers

**DOI:** 10.3389/fphar.2019.01533

**Published:** 2020-01-10

**Authors:** Gaozhi Chen, Yuyan Bao, Qiaoyou Weng, Yingxin Zhao, Xiaoyao Lu, Lili Fu, Lingfeng Chen, Zhiguo Liu, Xiaomin Zhang, Guang Liang

**Affiliations:** ^1^ Chemical Biology Research Center, School of Pharmaceutical Sciences, Wenzhou Medical University, Wenzhou, China; ^2^ Engineering Laboratory of Zhejiang Province for Pharmaceutical Development of Growth Factors, Biomedical Collaborative Innovation Center of Wenzhou, Wenzhou, China; ^3^ Department of Pharmacy, Sanmen People’s Hospital of Zhejiang, Sanmen, China; ^4^ Key Laboratory of Imaging Diagnosis and Minimally Invasive Intervention, The Fifth Affiliated Hospital of Wenzhou Medical University, Lishui, China

**Keywords:** NSCLC, EGFR, FGFR1, drug resistance, kinase inhibitor

## Abstract

In the past decades, epidermal growth factor receptor-tyrosine kinase inhibitors (EGFR-TKIs) had been proved as an effective treatment strategy for the patients with EGFR-mutated non-small-cell lung cancer (NSCLC). However, the tolerance for the EGFR-TKI always occurred after continuous administration for a period of time and limiting the application of these drugs. Activation of FGFR1 signaling pathway was one of the important escape mechanisms for EGFR-TKI resistant in NSCLC. Here, a novel dual inhibitor of EGFR^L858R/T790M^ and FGFR1, compound15c, was found and can efficiently overcame the EGFR-TKI resistance *via* its simultaneous inhibition of their kinase activities. Comparison with EGFR^L858R/T790M^ and FGFR1 inhibitor treatment alone or combined revealed that the inhibition of EGFR^L858R/T790M^ and FGFR1 activity by 15c was responsible for surmounting the intrinsic EGFR-TKI resistance in EGFR^L858R/T790M^-mutated H1975 cells and the acquired resistance in Afatinib-tolerant PC9 cells (AFA-PC9). Flow Cytometry and Caspase3 activity analysis assay showed that 15c induced significant the early apoptosis of H1975 cells. Xenograft tumor formation in BALB/c mice induced by a H1975 cells was suppressed by 15c treatment, with no changes in animal body weight. Generally, 15c may act as a new-generation EGFR-TKI for the therapy of NSCLC patients suffering a resistance to current TKI.

## Introduction

In the past few decades, to improve therapeutic activity and selectivity while developing antitumor drugs is always a great challenge. By targeting the genetic differences of cancer cells, several molecule-targeting drugs, such as Afatinib and Gefitinib, had been approved by U.S. FDA and shown their promising therapeutic activity against various cancers (Cataldo et al., 2011; Hirsch and Bunn, 2012). However, the therapeutic strategies of “one drug-hits-one target-treats-one disease” still face significant challenges as the drug resistance (To et al., 2019; Wojtaszek et al., 2019). Recent studies suggested that targeting the multiple targets might be a practicable approach to ameliorate the therapeutic activity and selectivity, meanwhile preventing the drug resistance (To et al., 2019).

Among cancer-related deaths, lung cancer is the leading cause that contributed to 27% of cancer-related mortality (Quintanal-Villalonga et al., 2019) and almost 85% of lung cancers were identified as non-small cell lung cancer (NSCLC) (Chang, 2011). The high dynamic of epidermal growth factor receptor (EGFR), one of the classic receptor tyrosine kinase (RTK), in NSCLC (Hirsch et al., 2003) recommended it as an attractive target for the development of small-molecule tyrosine kinase inhibitors (TKIs) to treat with NSCLC. At the beginning, several first-generation EGFR-targeted TKIs, such as Erlotinib and Gefitinib, had been approved by U.S. FDA for the treatment of NSCLC patients that harbor activating mutations in the EGFR (L858R or delE746-A750) (Cohen et al., 2003; Cohen et al., 2005; Cataldo et al., 2011). However, resistance to those inhibitors can be acquired due to secondary mutations (Bean et al., 2007; Kuang et al., 2009). The most common secondary mutations in the EGFR is T790M at exon 20, which is named as “gatekeeper” mutation (Zhou et al., 2009). Subsequently, a series of second-generation and third-generation EGFR-TKIs had being developed to overcome the resistance, especially T790M-associated resistance (Riely, 2008; Niederst et al., 2015; Chen et al., 2017b). Unfortunately, tolerance still occurred as a second mechanism that activation of alternative pathways, such as cMet, IGF-1R, and FGFR (Kono et al., 2009). Unlike the widely reported cMet and IGF-1R, the FGFR-dependent signaling act as an escape mechanism for EGFR-TKI resistance still have long way to go (Kono et al., 2009).

Fibroblast growth factor receptors (FGFRs) are a family of RTKs with four different members (FGFR1–4), and each of the FGFR contain an extracellular immunoglobulin domains, a single transmembrane domain and an intercellular kinase domain (Li, 2019). Moreover, genetic abnormalities or aberrant activation of the FGFR signal transduction have been implicated with the pathogenesis of the various disease and disorders including cancer, Alzheimer’s disease, diabetes and its complications (Ferrer and Marti, 1998; Gozgit et al., 2012; Harrison, 2012; Gallagher and LeRoith, 2013; Touat et al., 2015; Li, 2019). Among these receptors, FGFR1 amplification is identified in about 20% of NSCLC. In addition, Azuma et al., reported that the enhanced expression of FGFR1 conducted as an escape mechanism for cell survival of Afatinib-resistant cancer cells and may compensate the loss of EGFR-driven signaling pathway (Azuma et al., 2014). Further, Quintanal-Villalonga and his colleagues found that highly-expressed FGFR1 may result in a higher resistance to EGFR-TKI in the patients with EGFR-mutated lung cancer and those patients may benefit from combined EGFR/FGFR inhibition (Quintanal-Villalonga et al., 2019). Raoof and her colleagues performed whole-genome CRISPR screening and identified FGFR3 as the top target facilitating the survival of mesenchymal EGFR mutant cancers (Raoof et al., 2019). Therefore, dual EGFR-FGFR1 blockade may be a promising clinical strategy to overcome EGFR-mutated NSCLC.

Nowadays, drugs with polypharmacological activities are shown to be advantageous over combination therapy as their lower incidences of side effects and more resilient therapies (Anighoro et al., 2014). Thus, in the current study, we find that compound 15c, an EGFR^L858R/T790M^ selective inhibitor in our previous study (Chen et al., 2017a), exhibited a dual inhibitory activity against EGFR^L858R/T790M^ and FGFR1 and efficiently overcame the EGFR-TKI tolerance in EGFR-mutated NSCLC cells *via* concurrently inhibiting these two kinases activity. We suggest that 15c may act as a new-generation EGFR-TKI for the therapy of NSCLC patients suffering a resistance to current TKI.

## Materials and Methods

### Cell Culture and Reagents

WZ4002 (#S1173) and AZD4547 (#S2801) were purchased from Selleck Chemicals. Human bronchial epithelial cell line BEAS-2B, human lung squamous cancer cell line H520, and human NSCLC cell lines H1975 and PC9 were procured from the Institute of Biochemistry and Cell Biology, Chinese Academy of Sciences and tested for mycoplasma contamination by DAPI staining before experiment. All the cells were maintained in RPMI-1640 medium (#C11875500BT, Gibco) with 10% FBS (#10270-106, Gibco), 100 μg/ml streptomycin, and 100 U/ml penicillin (#15140122, Gibco) and placed in a humidified cell incubator (5% CO_2_, 37°C). Antibodies including anti-p-EGFR (#3777S), anti-p-FGFR1 (#2544S), anti-EGFR (#2646S), anti-FGFR (#9740S), anti-GAPDH (#5174S), and HRP-linked anti-rabbit IgG (#7074S) were purchased from Cell Signaling Technology (Danvers, MA, USA).

### Kinase Inhibition Assay

The kinase inhibitory activities of candidate and positive inhibitors were tested *via* a Caliper Mobility Shift Assay. The difference between substrate and its phosphorylated product was detected to characterize the activity. Shortly, the reaction solution containing compounds, substrates, ATP, and enzymes was mixed well and transferred to a 384-well plate for the experiment. EDTA was introduced to terminate the process after incubate for 1h at room temperature. The data was collected on an EZ Reader II (Caliper Life Sciences, MA). The inhibitory rates of tested compounds were calculated depending on the negative control wells (without ATP) and positive control wells (without compounds). The recombinant kinases, including EGFR^WT^ (#08-115), EGFR^L858R/T790M^ (#08-510), and FGFR1^WT^ (#08-133) were acquired from Carna Biosciences (Kobe, Japan). All the independent experiments were executed in duplicate and three times at six concentrations (0.001, 0.01, 0.1, 1, 10, and 100 μM) and IC_50_ value was calculated.

### Anti-Proliferation Assay (MTS Assay)

All kinds of cells (4×10^3^) were planted in 96-well plate and cultured overnight before examination. The protocol was formulated according to CellTiter 96^®^ AQ_ueous_ One Solution Cell Proliferation Assay (MTS) Technical Bulletin. Briefly, after treated the cells with different compounds for 72 h, 20 μl CellTiter 96^®^ AQ_ueous_ One Solution Reagent (MTS, #G3580, Promega, San Luis Obispo, CA) was added and the system was incubated for another 4 h at 37°C. The absorbance at 490 nm was recorded by using a microplate reader (SpectraMax M2, Molecular Devices, Sunnyvale, CA). The results of three independent assays were exhibited as IC_50_ value (mean ± SEM).

### Western Blot Analysis

After treated with compounds, cells or tumor tissues were harvested and lysed in protein lysate buffer followed by centrifugation (12,000 rpm, 10 min, 4°C), supernatants were collected. The protein concentrations were measured using the Quick Start™ Bradford Protein Assay Kit (#5000201, Bio-Rad, Hercules, CA). Equivalent amount of protein samples were separated by 12% SDS-polyacrylamide gel (SDS-PAGE) and then transferred to PVDF membrane. The blotting was blocked with 5% nonfat milk at room temperature for 2 h and then incubated with primary antibody at 4°C for overnight. At last, anti-rabbit HRP-conjugated secondary antibody was added and incubated with membrane for 1 h. Between every two steps, the membrane will be washed with TBST for three times. The immune-reactive bands were detected *via* Clarity Max Western ECL Substrate Kit (#1705062, Bio-Rad, Hercules, CA). The density of the immune-reactive bands was analyzed by Image J (National Institute of Health, MD).

### Cell Apoptosis Analysis

H1975 cells were seeded on 60-mm dishes for 12 h, and then treated with DMSO (vehicle), 15c (0.5, 2.5, 5, or 10 μM), WZ4002 (10μM), AZD4547 (10μM), or WZ4002 (10 μM) combined with AZD4547 (10 μM) for 24 h. After harvested and washed with PBS, cells were double stained with FITC Annexin V Apoptosis Detection Kit I (#556547, BD Biosciences, CA). The apoptosis were evaluated by using a FACSCalibur flow cytometer (BD Biosciences, CA).

### Analysis of Caspase-3 Activity

The activity of Caspase-3 in H1975 cell lysates was detected by using a Caspase-3 Activity Kit (C1115, Beyotime Institute of Biotechnology, Nantong, China). The caspase-3 activity was normalized and calculated as percentage of control group.

### Establishment of Afatinib-Resistant PC9 Cells

PC9 cells cultured in RPMI 1640 with 10% FBS were continuously exposed to Afatinib at the stepwise increased concentrations up to 1 µM over the following 3 months. After that, cells remained were cultured in Afatinib-free growth medium until the stable growth was restored. The isolated Afatinib-resistant cell line was named as AFA-PC9.

### *In Vivo* Antitumor Study

Protocols for animal studies were approved by the Wenzhou Medical University Animal Policy and Welfare Committee. Male BALB/c nu/nu mice (6 weeks old, 18–22 g) were acquired from Vital River Laboratory Animal Technology Co., Ltd. (Beijing, China). Animals were housed at a constant room temperature with a 12 h: 12 h light/dark cycle and fed with a standard rodent diet and water. H1975 cells were injected subcutaneously into the right flank (1×10^7^ cells in 200 μl of PBS). 40 mice were divided into five groups (n = 8/group): control (vehicle), 15c (10 mg/kg/day), WZ4002 (10 mg/kg/day), AZD4547 (10 mg/kg/day), and WZ4002 (10 mg/kg/day) combined with AZD4547 (10 mg/kg/day). The compounds were given by intraperitoneal (i.p.) injection. The tumor volumes were determined at the indicated time points (V = 0.5 × L × W^2^; L: length, W: width, V: volume). After sacrificed, the tumors were weighed and collected for studies on the protein expression.

### Statistical Analysis

At least three independent experiments were performed for each assay (n≥3). Data were shown as mean ± SEM. GraphPad Prism 5.0 (GraphPad, San Diego, CA) was used for the statistical analyses. Student’s *t*-test and two-way ANOVA were employed to analyze the differences between different sets of data. *P* < 0.05 was considered statistically significant and exhibited by * or # characters.

## Results

### Compound 15c Acted as an EGFR^L858R/T790M^/FGFR1 Dual Inhibitor and Selectively Kills Human Lung Cancer Cells

**Figure 1:**
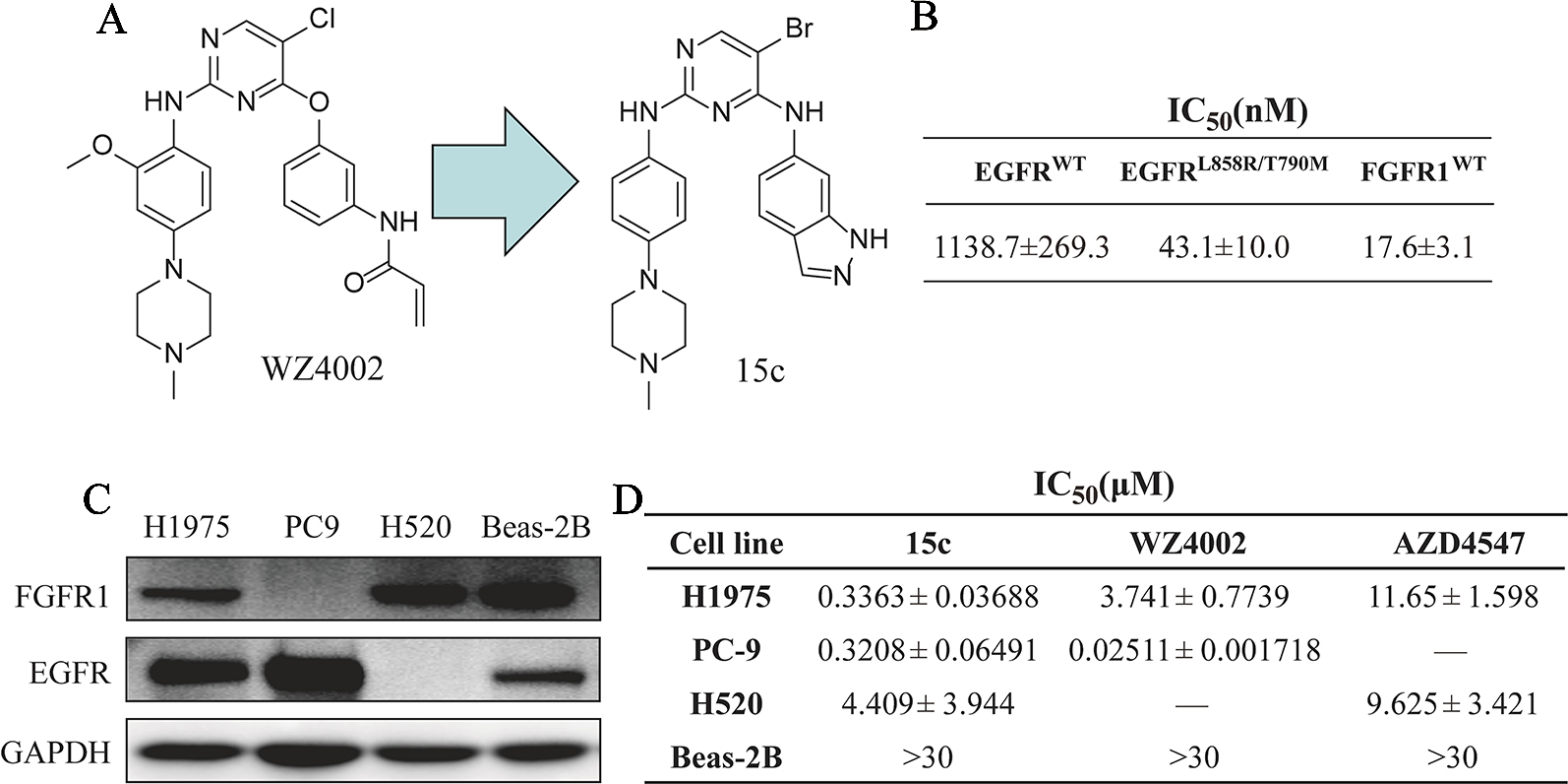
Compound 15c acted as an EGFR^L858R/T790M^/FGFR1 dual inhibitor and selectively kills human lung cancer cells. **(A)** The chemical structure of WZ4002 and 15c. **(B)** The kinase inhibitory activity against EGFR^WT^, EGFR^L858R/T790M^, and FGFR1^WT^ of 15c. **(C)** Immunoblots of the expression levels of EGFR and FGFR1 protein in H1975, PC9, H520, and Beas-2B cell lines. **(D)** Anti-proliferation effect of 15c against H1975, PC9, H520, and Beas-2B cell lines. The IC_50_ values were reported as μM values (n = 3 independent experiments).

In our previous work (Chen et al., 2017a), we designed and optimized a series analogue of WZ4002 and evaluated their inhibition activity against EGFR^L858R/T790M^, and found that several derivatives exhibited a high potency and selectivity between EGFR^L858R/T790M^ and EGFR^WT^. In order to explore whether those compounds can inhibit the FGFR1 kinase activity, all the compounds were measured the kinase inhibitory effect against EGFR^WT^, EGFR^L858R/T790M^, and FGFR1^WT^ in a cell-free system called Caliper Mobility Shift Assay. As shown in **Figures 1A, B**, 15c exhibited the high inhibition towards EGFR^L858R/T790M^ and FGFR1^WT^, and relatively lower inhibition against EGFR^WT^. Thus, 15c can act as an EGFR^L858R/T790M^ and FGFR1^WT^ dual inhibitor.

Kinases such as EGFR and FGFR play a critical role in the proliferation of cancer cells. Thus, we next detected the anti-proliferation effects of 15c on cultured human lung cancer cells and normal cells. Four cell lines, lung cancer PC9 cells (EGFR^L858R+^ and FGFR1^−^), lung cancer H520 cells (EGFR^−^ and FGFR1^+^), lung cancer H1975 cells (EGFR^L858R/T790M+^ and FGFR1^+^), and normal lung epithelial cells Beas-2B cells (EGFR^+^ and FGFR1^+^), were chosen for the cytotoxic assay (**Figure 1C**). 15c exhibited high anti-proliferation activity against lung cancer cells rather than normal Beas-2B cells (highest concentration at 30 μM) (**Figure 1D**). Those findings indicated that 15c acted as an EGFR^L858R/T790M^/FGFR1 dual inhibitor in cell-free system and selectively inhibited the proliferation of human lung cancer cells.

### Compound 15c Inhibited the Phosphorylation of EGFR and FGFR1 in the Cell System

**Figure 2:**
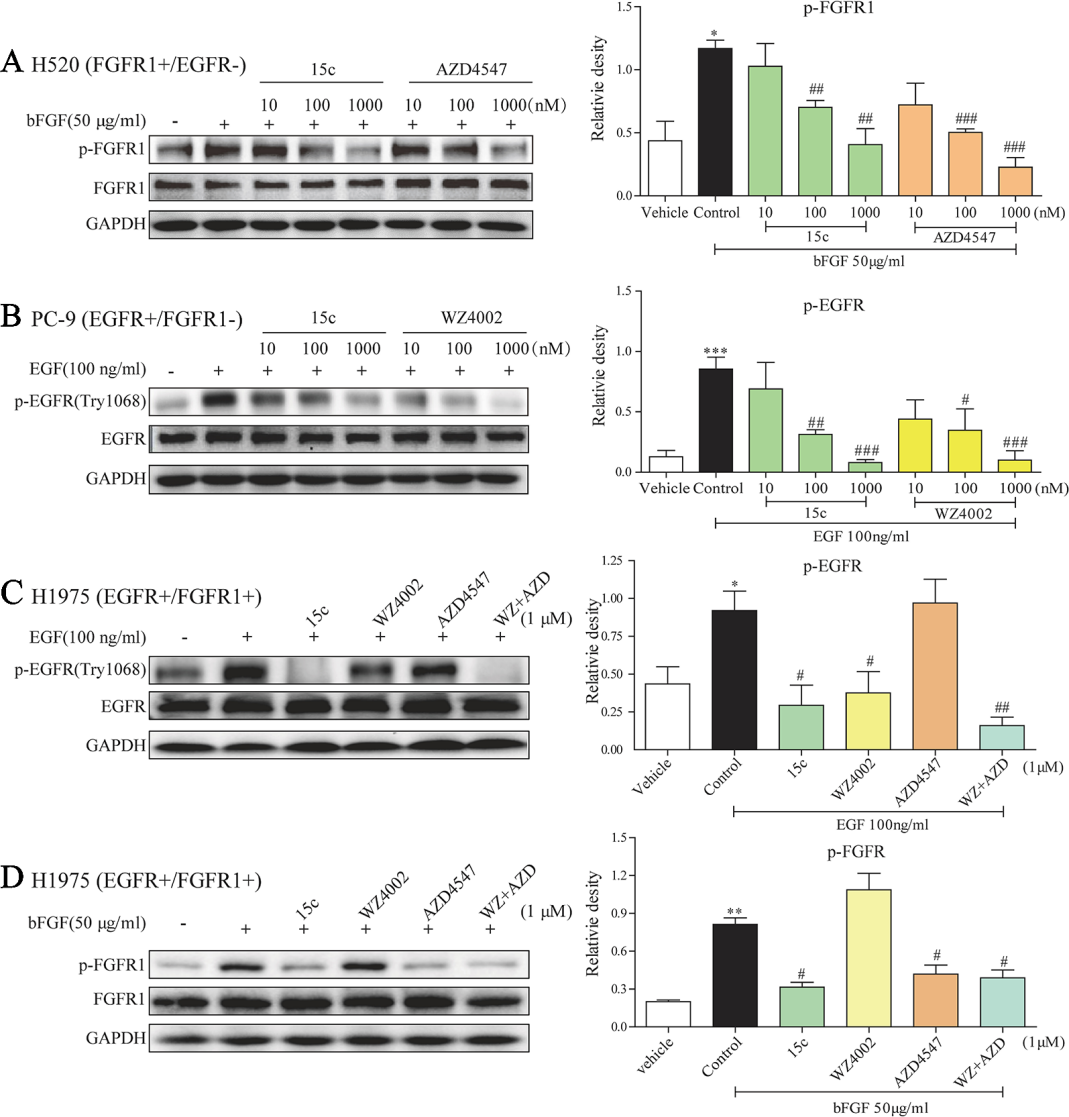
Compound 15c inhibited the phosphorylation of EGFR and FGFR1 in the cell system. **(A**–**D)** Representative immunoblots of phosphorylated FGFR1/EGFR, total FGFR1/EGFR, and GAPDH (sample loading controls) in three different cell lines that treated with inhibitors (n = 3 independent experiments for each panel). **(A)** Analysis of the p-FGFR1 level in bFGF-stimulated H520 cells. **(B)** Analysis of the p-EGFR level in EGF-stimulated PC9 cells. **(C)** Analysis of the p-EGFR level in EGF-stimulated H1975 cells. **(D)** Analysis of the p-FGFR1 level in bFGF-stimulated H1975 cells. The statistical results were shown as means ± SD (n = 3) on the right of each panels. Significant difference was indicated as * or # character (*p < 0.05, **p < 0.01, and ***p < 0.001 versus vehicle group, ^#^p < 0.05, ^##^p < 0.01, and ^###^p < 0.001 versus control group).

In order to further confirm whether compound 15c can impede the phosphorylation of EGFR and FGFR1 in cells, the kinase inhibitory activity of 15c was detected in various ligand stimulated cancer cells. As mentioned before, PC9 is a human lung adenocarcinoma cell line with EGFR^L858R^ mutation without FGFR1 expression, while H520 is a human lung squamous cell carcinoma cell line with the high expression of FGFR1 but without EGFR expression. Thus, the p-EGFR levels in EGF-induced PC9 cells and the p-FGFR1 levels in FGF-stimulated H520 cells were determined, respectively. The third-generation EGFR-TKI WZ4002 and the FGFR1-TKI AZD4547 were selected for comparison. The results were summarized in **Figures 2A, B**. Compound 15c, similar to the positive control, can inhibit the phosphorylation of FGFR1 and EGFR in a dose-dependent manner in different cell lines.

In addition, H1975 is a human adenocarcinoma cell line with EGFR^L858R/T790M^ mutation and FGFR1 expression, which can be used as an EGFR-TKI acquired resistant cell line. In this part, we treated the H1975 cells with 1 μM 15c, WZ4002, AZD4547, or WZ4002 and AZD4547 combination followed by bFGF or EGF stimulated. The p-FGFR1 and p-EGFR levels were determined by Western blot. In the EGF-stimulated H1975 cells (**Figure 2C**), 15c and WZ4002 exhibited the similar inhibitory activity against the phosphorylation of EGFR and AZD4547, a FGFR1 inhibitor, showed no effect on the phosphorylation of EGFR. The inhibitory effect of WZ4002 and AZD4547 combination was slightly better than 15c or WZ4002 alone, but no significant difference was noted. Similarly, in the bFGF-induced H1975 cells (**Figure 2D**), 15c and AZD4547 exhibited the comparable inhibitory activity against the phosphorylation of FGFR1 and WZ4002 showed no effect. The inhibitory impact of WZ4002 and AZD4547 combination was almost the same to 15c or WZ4002 alone. Those results revealed that 15c was a potential EGFR^L858R/T790M^/FGFR1 dual inhibitor in the cell system.

### Compound 15c Induced Caspase-Dependent Apoptosis in H1975 Cells

**Figure 3:**
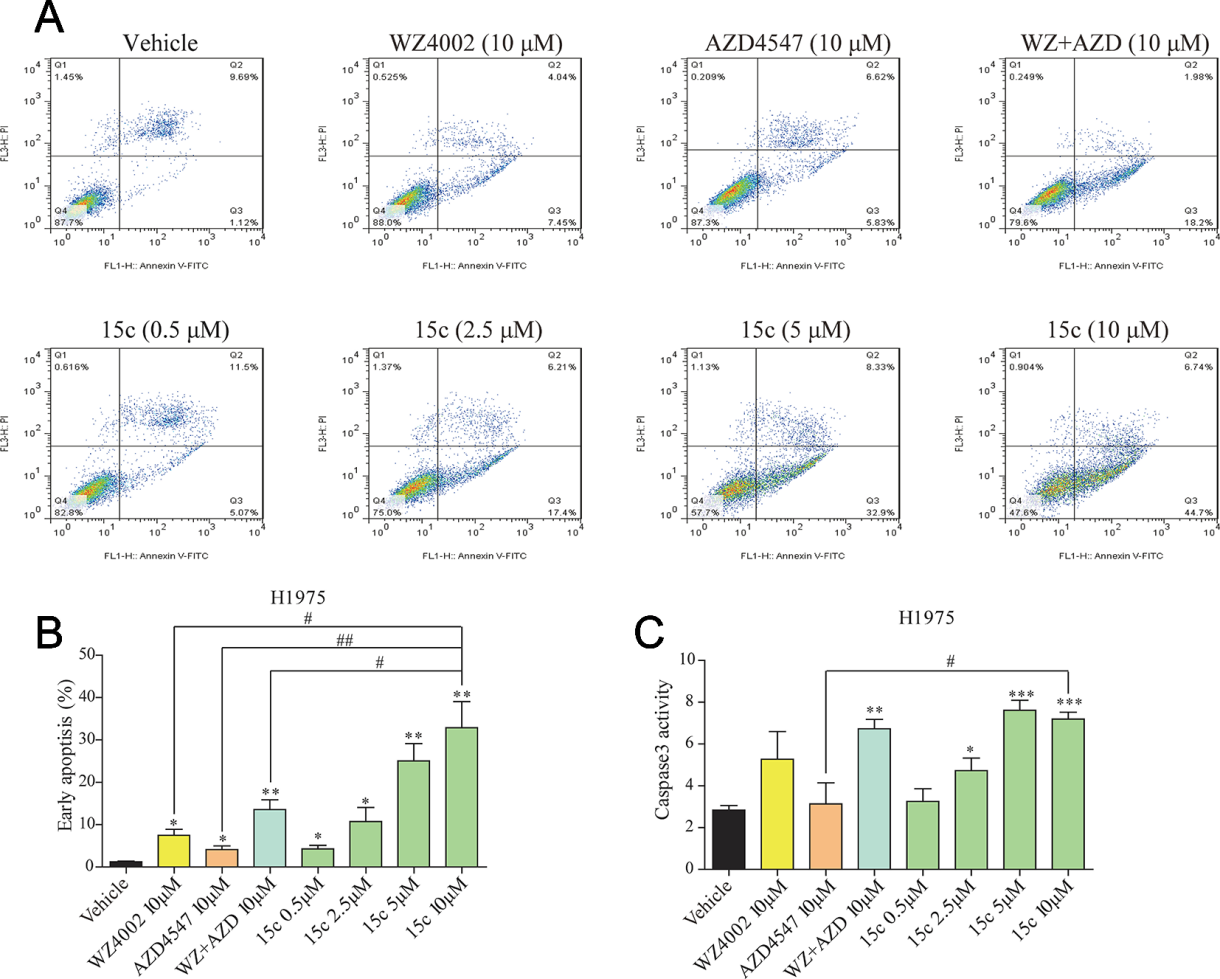
Compound 15c induced caspase-dependent apoptosis in H1975 cells. **(A)** Apoptosis induced by inhibitors treatment in H1975 cells. Cells were incubated with the indicated doses of 15c, WZ4002, AZD4547, and WZ4002 + AZD4547 for 24 h, and then harvested and analyzed by flow cytometry. Representative data from one experiment are shown. **(B)** The percentage of apoptotic cells in the treatment groups was calculated. **(C)** Caspase-3 activity of H1975 cells treated with 15c (0.5, 2.5, 5, 10 μM), AZD4547 (10 μM), WZ4002 (10 μM), or AZD4547 (10 μM) + WZ4002 (10 μM) for 24 h. All the statistical results are shown as means ± SD (n = 3). *p < 0.05, **p < 0.01, and ***p < 0.001 versus vehicle group, ^#^p < 0.05 and ^##^p < 0.01 versus 10 μM 15c group.

To explore whether 15c can accelerate H1975 cells apoptosis, a series apoptosis related experiments were introduced. H1975 cells that starved for 12 h were treated with variable level of test compounds, positive inhibitors, or vehicle for 24 h. After harvested and washed with PBS, cells were double stained with FITC Annexin V Apoptosis Detection Kit I. The apoptotic cells were detected by flow cytometry analysis and the results were also illustrated in the column chart (**Figures 3A, B**). Significant apoptosis was observed in 15c treated H1975 cells in a dose-dependent manner compared with the vehicle group. Moreover, the apoptosis rates of 15c at 5 μM and 10 μM were much higher than that WZ4002 or AZD4547 treated alone or combined at 10 μM. In addition, caspase 3 activity assay were further applied to verify the effect of 15c on inducing H1975 apoptosis (**Figure 3C**). The results suggested that 15c could induced the apoptosis of H1975 cells *via* activating caspase-dependent apoptosis.

### Compound 15c Overcame Afatinib-Tolerant in Established Acquired Resistant PC9 Cell Line

**Figure 4:**
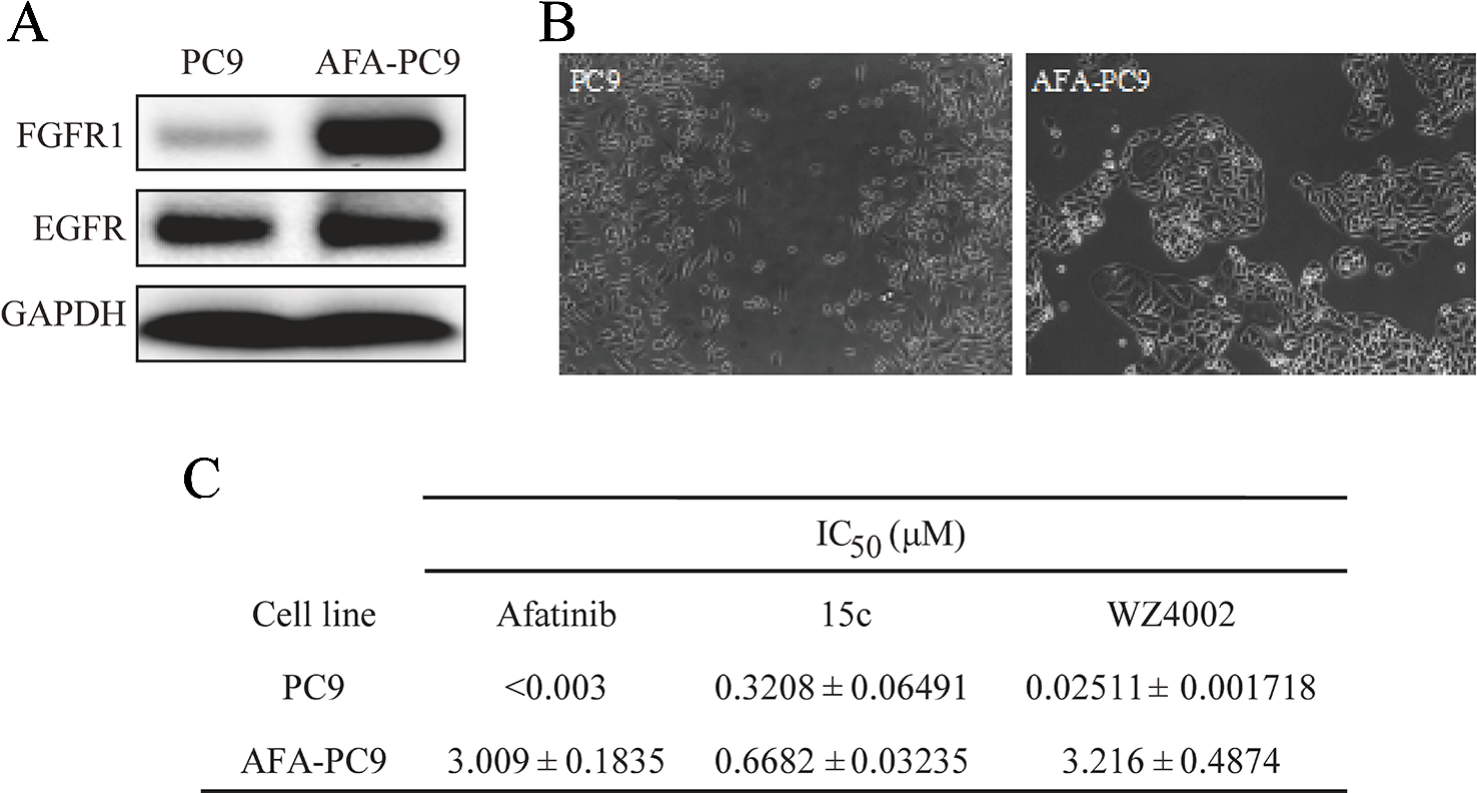
Compound 15c overcame Afatinib-tolerant in established acquired resistant PC9 cell (AFA-PC9). Cancer cell line PC9 was continuously exposed to Afatinib at stepwise increased concentrations up to 1 µM over the following 3 months to establish an Afatinib-resistant PC9 cell line named ADA-PC9. **(A)** Western Blot analysis of the EGFR and FGFR1 expression in PC9 cells and AFA-PC9 cells. **(B)** Morphological changes of AFA-PC9 cells compared to PC9 cells; **(C)** Anti-proliferation effect of 15c, WZ4002, and Afatinib against PC9 and AFA-PC9 cell lines. The IC_50_ values were reported as μM values.

The effect of 15c on reversing drug resistant in lung cancer cells was further studied by domesticated a cell line resistant to Afatinib. The PC9 cells were continuously exposed to Afatinib at the stepwise increased concentrations from 0.025 to 1 µM over the following 3 months to construct the Afatinib-resistant cell lines AFA-PC9. Western blot analyses of PC9 and AFA-PC9 were shown in **Figure 4A**, and the FGFR1 expression was significantly amplified in AFA-PC9 and the EGFR expression was not affected. The morphology change of the AFA-PC9 was demonstrated in **Figure 4B**. Compared to PC9, AFA-PC9 cells started to grow in clusters, which was completely consistent with the conclusions in previous studies. MTS analysis was also carried out on the drug-resistant AFA-PC9 (**Figure 4C**). Compared with the PC9 cells, the sensitivity of AFA-PC9 cells to Afatinib was reduced by more than 1000 times. Then, we compared the anti-proliferative effects of compound 15c and WZ4002 on AFA-PC9 and PC9 cells. It turned out that the sensitivity of AFA-PC9 to WZ4002 decreased more than 100 times (IC_50_ was changed from 0.025 to 3.2 μM) while only decreased two times against 15c (IC_50_ was changed from 0.32 to 0.66 μM), indicating that 15c can overcame the acquired resistant to Afatinib in PC9 cell line.

### Compound 15c Attenuated H1975 Xenograft Tumor Growth *In Vivo* by Suppressing p-EGFR and p-FGFR1 Levels

**Figure 5:**
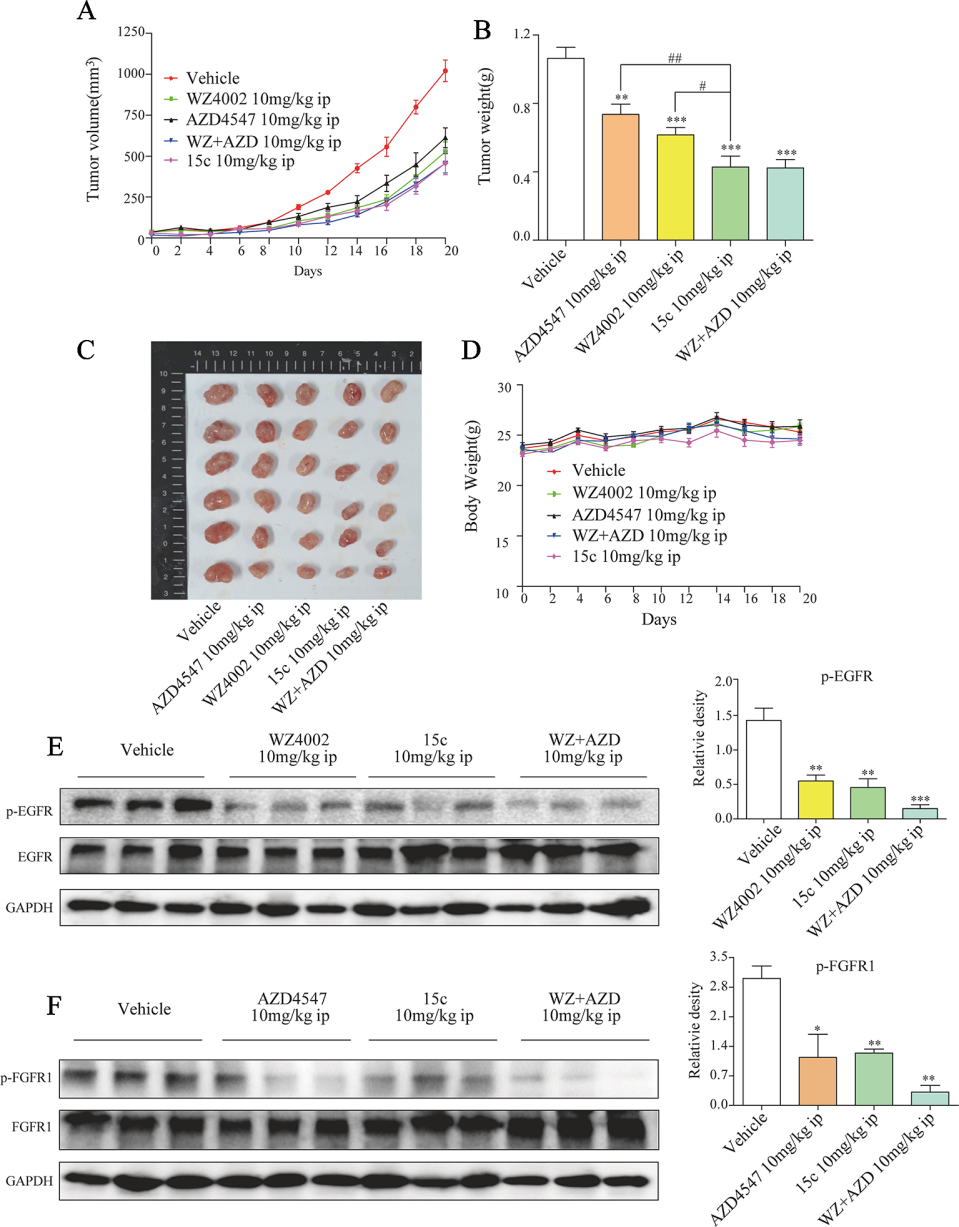
Compound 15c inhibited H1975 xenograft tumor growth *in vivo*, accompanied with decreased p-EGFR and p-FGFR1 levels. Tumor-bearing mice were intraperitoneally injected with 15c (10 mg/kg), WZ4002 (10 mg/kg), AZD4547 (10 mg/kg), or WZ4002 (10 mg/kg) + AZD4547 (10 mg/kg). **(A**–**C)** Tumor volume and tumor weight of H1975 human lung cancer xenografts in nude mice. **(D)** Body weight was measured every 2 days. Points, means of seven mice; bars, SEM. **(E**, **F)** Analysis of pFGFR1 and p-EGFR levels in tumor tissues. *p < 0.05, **p < 0.01, and ***p < 0.001 versus vehicle group, ^#^p < 0.05 and ^##^p < 0.01 versus 10 mg/kg 15c group. ip, intraperitoneally injected.

Subcutaneous xenograft model of H1975 cells in immunodeficient mice was applied to evaluate the *in vivo* anti-tumor activity of 15c. Male BALB/c nu/nu mice (6 weeks old, 18–22 g) were subcutaneously injected with H1975 cells (1×10^7^ cells in 200 μl of PBS). When the tumor volume was around 80–100 mm^3^, the mice were randomly divided into five groups (eight in each) including negative control group (Vehicle group), 15c group (10 mg/kg/day, ip), WZ4002 group (10 mg/kg/day, ip), AZD4547 group (10 mg/kg/day, ip), and WZ4002 combined with AZD4547 group (10 mg/kg/day, ip). After intraperitoneal administration of compound for 20 days, the tumor volume and weight of each group were measured and shown in **Figures 5A–C**. 15c significantly reduced H1975 tumor volume and weight versus vehicle control. Even compared with the positive inhibitor alone, 15c still exhibited a better tumor-inhibition effect. Only WZ4002 and AZD4547 combination administration can restrain the H1975 tumor to the same size of the tumor treated by 15c. Importantly, these is no significant weight loss of mice even treated 15c for 20 days (**Figure 5D**). Mechanistically, western blot analysis of tumor tissues revealed that 15c attenuated H1975 xenograft tumor growth *in vivo* by suppressing the phosphorylation of FGFR1 and EGFR (**Figures 5E, F**).

## Discussion

Lung cancer is the leading cause of cancer-related death worldwide with NSCLC accounting for 85% of cases. Therefore, it is of great theoretical and practical significance to study the pathogenesis of lung cancer and develop the corresponding therapeutic drugs. In the past few decades, it has been determined that the activation of EGFR is closely related to the genesis and progress of lung cancer (Lynch et al., 2004; Paez et al., 2004; Pao et al., 2004; Sharma et al., 2007). Thus, EGFR has attracted great attentions as it is an ideal target for the cancer therapy. There are a series of small molecular inhibitors that can inhibit the kinase activity of EGFR had been found and named as EGFR-TKIs (Sequist et al., 2007; Pao and Chmielecki, 2010). Gefitinib and Erotinib are the first-generation EGFR-TKIs that have been studied clinically with a great advance. However, patients generally develop secondary mutations, such as T790M gatekeeper, and lead to drug resistance after EGFR-TKIs treated for 12 months (Chen et al., 2017b). As a result, second and third-generation EGFR-TKIs, e.g. Afatinib, WZ4002, and Osimertinib, are developed to overcome the T790M mutation-associated resistance (Zhou et al., 2009; Mok et al., 2017). The poor therapeutic window of Afatinib limited its clinical application (Chen et al., 2017b). Osimertinib exhibits an excellent treatment effect for NSCLC patients with EGFR T790M-mutated, but acquired resistance is still inevitable (Oxnard et al., 2018). Thus, there is still an urgent demand to establish an effective antitumor strategy for those NSCLC patients with acquired resistance to second or third-generation EGFR-TKIs.

Müller-Tidow and his colleagues identified the expression of 56 RTKs in primary NSCLC tumors and found that 33 RTKs are expressed at the mRNA level in 25% samples (Müller-Tidow et al., 2005), indicating that those RTKs may function as an alternatives to EGFR signal pathway in NSCLC, such as cMet, Axl, IGF-1R, and PDGFR. The amplification of cMet is a major factor of acquired resistance in lung cancer and the combination of Gefitinib and cMet antibody can significantly enhance the growth inhibition in cMet over-expressing cell lines (Bean et al., 2007; Zucali et al., 2008). Similarly, the IGF-1R survival pathway contribute to Gefitinib resistance and synergistically inhibition of IGF-1R could strengthen the anti-tumor effect of Gefinitib (Desbois-Mouthon et al., 2006). In addition, Axl lead to EGFR-TKIs resistant by increasing expression and forming a hetero-dimerization with EGFR and inhibition of Axl could prevent or overcome acquired resistance to EGFR-TKIs in EGFR-mutated lung cancer (Zhang et al., 2012; Vouri et al., 2016). Recently, several researches mentioned that the activation of the autocrine ring of FGF/FGFR, one of the typical RTKs, could act as a compensatory mechanism to promote the survival and growth of EGFR-TKIs resistant lung cancer cells. These findings declared that simultaneous inhibition of EGFR and FGFR1 activity may be a novel and effective strategy to overcome FGFR1-related EGFR-TKIs resistant in NSCLC.

Nowadays, combination therapy of various drugs with different targets has become an important strategy for cancer treatment (Bell, 2013). Combined drugs can improve the curative effect as the synergism of different activity. However, increased off-target effect and metabolic pressure restricted the application of combined administration. Moreover, drugs with polypharmacological activities are shown to be advantageous over combination therapy as their lower incidences of side effects and more resilient therapies. Thus, developing dual-inhibitors that target FGFR1 and EGFR will be an efficient way to reverse FGFR1-related EGFR-TKIs resistant in NSCLC.

Here, we presented an EGFR^L858R/T790M^/FGFR1 dual-inhibitor 15c, which can simultaneous inhibited the kinase activity of EGFR^L858R/T790M^ and FGFR1 both in kinase assay and cell study, for the treatment of FGFR1-amplified EGFR-TKIs resistant lung cancers. The anti-proliferation effects of 15c on NSCLC cell lines and normal BEAS-2B cell line showed that 15c could effectively hold back the proliferation of NSCLC cells rather than normal cells. Particularly, 15c exhibited a better inhibitory effect to H1975 cells and AFA-PC9 cells, which contained EGFR mutation and FGFR1 overexpression, than WZ4002 and AZD4547. Further flow cytometry and Caspase 3 activity analysis demonstrated that 15c could induce the apoptosis in a dose-dependent manner and the effect was better than WZ4002 combined AZD4547 treatment. Finally, the *in vivo* study as well revealed that the anti-tumor ability of 15c was comparable to the combination administration of WZ4002 and AZD4547 but better than the separate administration.

In summary, acquired resistant is the major reason for EGFR-TKI tolerant NSCLCs and FGFR1 amplification has been proved that contribute to the EGFR-TKI resistant. Thus, EGFR^L858R/T790M^/FGFR1 dual inhibitor 15c can be developed as an ideal candidate drug for FGFR1-amplified EGFR-TKIs resistant lung cancers.

## Data Availability Statement

The raw data supporting the conclusions of this article will be made available by the authors, without undue reservation, to any qualified researcher.

## Ethics Statement

The animal study was reviewed and approved by the Wenzhou Medical University Animal Policy and Welfare Committee.

## Author Contributions

Participated in research design: GC, QW, GL. Cell and animal study: GC, YB, QW, YZ, and XL. Kinase study: LF. Performed chemical synthesis of 15c: LC and ZL. Contributed to the writing of the manuscript: GC, XZ, GL.

## Funding

This work was supported by the National Key R&D Program of China (2017YFA0506000), the National Natural Science Foundation of China (Grants No. 81703352 and 81773579), the Zhejiang Province Natural Science Foundation (LY17B020008), the Zhejiang Medical Health Technology Platform Project (2018KY183) and the Zhejiang Natural Science Foundation Pharmaceutical Society Joint Fund Project (LYY19H310004).

## Conflict of Interest

The authors declare that the research was conducted in the absence of any commercial or financial relationships that could be construed as a potential conflict of interest.
